# Anti-synthetase Syndrome: A Diagnostic Dilemma

**DOI:** 10.7759/cureus.36760

**Published:** 2023-03-27

**Authors:** Muniba Aslam, Sulhera Khan, Wajeeha Batool, Zeeshan Ali, Iqra M Hanif

**Affiliations:** 1 Internal Medicine, Jinnah Postgraduate Medical Centre, Karachi, PAK; 2 Gastroenterology, Jinnah Postgraduate Medical Centre, Jinnah Sindh Medical University, Karachi, PAK; 3 Dermatology, Jinnah Postgraduate Medical Centre, Karachi, PAK

**Keywords:** polymyositis, non-erosive arthritis, anti-jo1 antibodies, inflammatory myopathy, anti-synthetase syndrome

## Abstract

Among the various inflammatory myopathies, the anti-synthetase syndrome (ASS) is a rare entity with autoantibodies directed against aminoacyl-transfer ribonucleic acid synthetase. Its clinical spectrum ranges from myopathy and non-erosive arthritis to dyspnea and cough of pulmonary interstitial disease and from hyperkeratotic skin changes to spasms of blood vessels causing Raynaud’s phenomenon. We present a case of a 21-year-old female who had been suffering from fever, night sweats, and weight loss for two years and had remained undiagnosed. She came to our hospital with new-onset muscle weakness, small joint arthralgia, and skin changes. Physical examination showed inflammation involving multiple small joints and characteristic hyperkeratotic skin changes in the distal and lateral phalanges of the hands and feet. Raised creatine phosphokinase levels indicated the possibility of myositis along with positive anti-nuclear antibodies, suggesting an autoimmune rheumatic disorder. Inflammatory myositis was later confirmed on biopsy. Further investigations revealed positive anti-Jo1 antibodies. The diagnosis of ASS was made despite the absence of pulmonary signs and symptoms. The patient was promptly started on prednisone and azathioprine. She showed some improvement in muscle weakness at the end of two months and continues to improve albeit slowly. Due to the lack of awareness about the rare disease among the non-rheumatologists, there was a significant delay in the patient's diagnosis. It is, therefore, important for primary care physicians to obtain a comprehensive history and perform a detailed clinical examination to make timely referrals to specialized healthcare professionals.

## Introduction

Anti-synthetase syndrome (ASS) belongs to the group of heterogenous inflammatory myopathies. Inflammatory myopathies are autoimmune disorders with specific autoantibodies [1]. Dermatomyositis and polymyositis are the most common diseases in the inflammatory myopathies group [2]. The autoantibodies seen in ASS are directed against eight different types of aminoacyl-transfer ribonucleic acid (tRNA) synthetases (ARS) [3]. Among these antibodies, it is seen that anti-Jo1 is the most prevalent [4]. ASS is closely related to dermatomyositis and polymyositis with interstitial lung disease and arthritis [2]. The prevalence is still unknown, and the disease more commonly affects females with a female-to-male ratio of 3:1 [5]. The literature suggests that human leukocyte antigen (HLA) DRB1*0301, DQA1*0501, and DQB1*0201 genes are common risk factors for the development of ASS [2].

The common clinical presentations of ASS, which include myositis, interstitial lung disease, arthritis, Raynaud’s phenomenon, and mechanic's hands [5], overlap with other more common autoimmune rheumatic diseases leading to diagnostic confusion. Some patients may also have symptoms of dry eyes, dry mouth, and difficulty swallowing, features overlapping with Sjogren’s syndrome and systemic sclerosis [6]. The patients may also have a history of non-specific features like long-term intermittent fever and weight loss [2]. Of the patients, 70-95% present with respiratory symptoms, dry cough, and shortness of breath in ASS [2]. Therefore, pulmonary involvement is a prominent component of the disease presentation, and also the severity and type of pulmonary involvement determine the severity of the disease, often also seen in most case reports as the primary complaint. However, myositis is seen in >90% of the patients resulting in proximal muscle weakness, raised serum creatine phosphokinase (CPK), and specific muscle biopsy findings [1].

We present a case of a young female with predominantly myositis alongside the constitutional symptoms of fever, weight loss, and pathognomonic mechanical hands. However, she lacked any pulmonary symptoms thereby producing a diagnostic dilemma clinically.

## Case presentation

A 21-year-old female was admitted to our ward with complaints of high-grade fever, drenching night sweats, weight loss, and gradually progressive symmetrical muscle weakness accompanied by pain for the last three months. The muscle pain and weakness had severely restricted her mobility such that she was unable to walk without support at the presentation. The power of the deltoid, biceps, and triceps was 3/5 while the power of the flexors and extensors of the forearm verged on 4/5. Similarly, the power of the quadriceps and hamstrings was barely 3/5 with better performance of calf muscles at 4/5. Upon investigations, CPK levels were highly raised supporting the diagnosis of inflammatory myopathy. The erythrocyte sedimentation rate (ESR) was also raised. Aldolase levels were normal. The thyroid-stimulating hormone (TSH) was normal.

**Figure 1:**
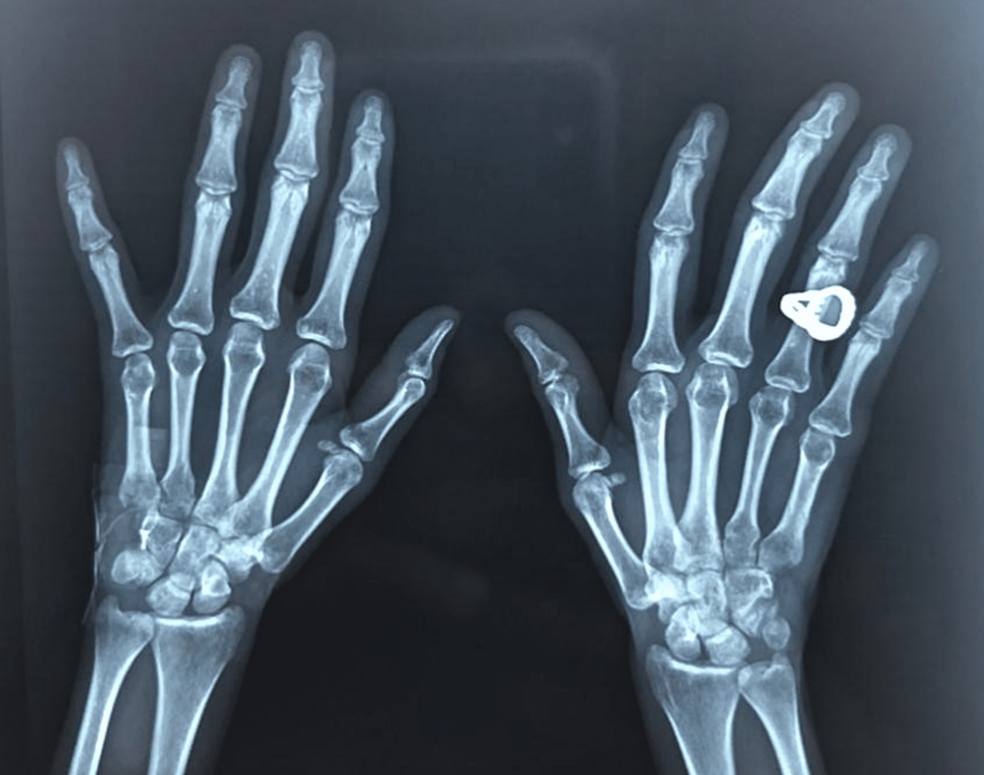
Hand X-ray X-ray showing normal symmetrical joints without bone overlapping except at the carpal and base of the metacarpal bones. No fractures, osteopenia, or bone deformities were noted.

Past history was significant for low-grade fever with drenching night sweats for the past two years alongside generalized myalgias and arthralgias with particular involvement of the small joints of the hand in the last six months. No history of morning stiffness, tenderness, warmth, or redness of the joints was given by the patient. She had taken multiple courses of antibiotics for the fever, which had failed to resolve. The metacarpophalangeal (MCP) and proximal interphalangeal (PIP) joints of the hands were erythematous, swollen, and mildly tender on palpation but the rest of the joints did not show signs of inflammation. The rheumatoid factor was positive with negative anti-cyclic citrullinated peptide (anti-CCP) antibodies. As the rheumatoid factor had been previously positive as well, the patient had been prescribed non-steroidal anti-inflammatory drugs (NSAIDs) multiple times. However, radiological imaging failed to show any features of arthritis, as seen in Figure 1.

**Figure 2:**
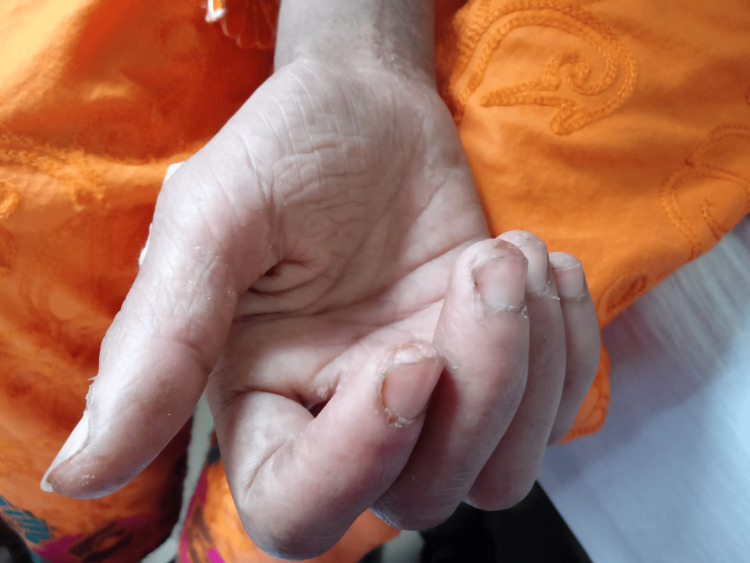
Mechanic's hands Image showing a nonpruritic, hyperkeratotic, and scaly eruption on the ulnar side of the thumb and radial side of other fingers, fingertips, and base of the nail bed.

A history of cutaneous eruptions in the form of red lines over the body was also given but without any physical evidence. Skin tightening, photosensitivity, and pruritis were denied. Cough and dyspnea were absent. The use of any prescription or recreational drugs was denied except over-the-counter analgesics like paracetamol and occasionally diclofenac and ibuprofen. During hand examination, she was found to have classic mechanic hands with hyperkeratosis of the tips of the fingers and palms, as seen in Figure 2.

**Figure 3:**
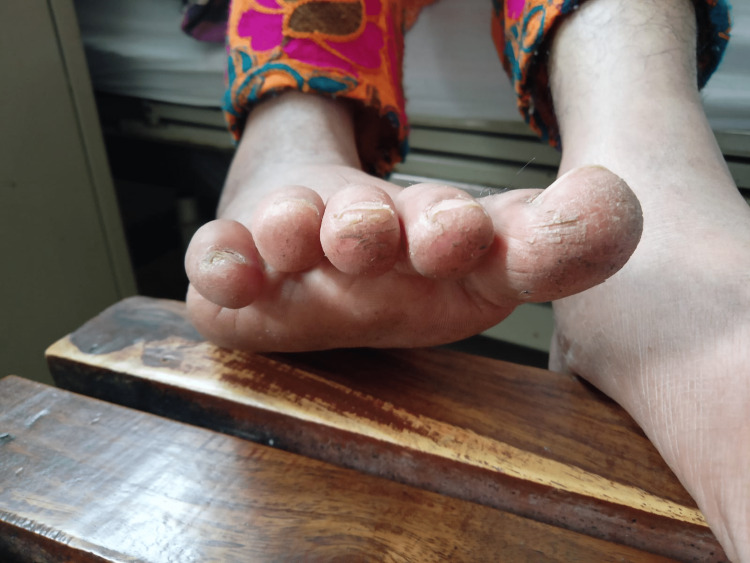
Hiker's feet Image showing hyperkeratosis and cracking of the skin of the toes.

Similar findings were also present in the feet consistent with the hiker’s feet, as in Figure 3.

Due to the patient's constitutional symptoms, we were initially concerned for disseminated tuberculosis due to its multifaceted presentation and prevalence in our community. It was excluded by a negative Mantoux test. A previous history of a positive hepatitis C test on indirect Coombs test (ICT) led us to consider cryoglobulinemia syndrome in our differential as well but the examination was negative for any type of peripheral neuropathy or purpura to support the diagnosis and polymerase chain reaction (PCR) for hepatitis C was undetectable. Complement levels (C3 and C4) were normal. Viral serology for human immunodeficiency virus (HIV) was negative.

The findings of myositis and a significantly raised CPK level together with positive anti-nuclear antibodies (ANA) led us to consider a rheumatological disorder and the department of rheumatology was taken on board. The combination of myositis and mechanic's hands was concerning for dermatomyositis but for the absence of prominent skin eruptions. A lack of any sign of interstitial lung disease on history, exam, or chest X-ray and the absence of Raynaud's phenomenon, both of which also form part of the diagnostic criteria, made us doubtful as to the presence of the rare ASS. However, an extended autoimmune profile revealed high titers of anti-Jo1 and anti-threonyl-tRNA synthetase (PL7) antibodies. The rheumatologist also requested electromyography (EMG) and muscle biopsy, which were subsequently performed and showed perifascicular necrosis, the characteristic finding of ASS. In EMG, abnormally short durations of the motor unit potential (MUP) patterns were seen in the deltoid and biceps and in the vastus lateralis. The patient was thus able to fulfill both the Connors and Solomon criteria for diagnosis of ASS due to the presence of anti-ARS antibodies associated with fever, mechanic's hands, and polymyositis.

Table 1 lists the investigations performed during the patient’s hospital stay.

**Table 1:** Investigations conducted during the hospital stay

Parameter	Result	Reference range
Hemoglobin (Hb)	12.6 g/dl	12.0-16.0
Hematocrit	34.6%	36-48
White blood cell (WBC) count	8.8*10^9 ^/L	5.0-10.0
Platelets	354*10^9^/L	140-400
Urea	22 mg/dl	10-50
Creatinine	0.24 mg/dl	0.5-1.5
Sodium	139 mEq/L	136-149
Potassium	3.9 mEq/L	3.8-5.2
Chloride	102 mEq/L	98-107
Total bilirubin	0.44 mg/dl	<1.0
Direct bilirubin	0.12 mg/dl	0-0.25
Alanine aminotransferase (ALT)	30 U/L	8-36
Aspartate aminotransferase (AST)	37 U/L	0-31
Alkaline phosphatase (ALP)	38 U/L	30-120
Gamma-glutamyl transferase (GGT)	10 U/L	10-31
Erythrocyte sedimentation rate (ESR)	37 mm/hr	0-25
C-reactive protein (CRP)	16.7 mg/dl	<5.0
Creatine phosphokinase (CPK)	8,000 mcg/L	10-120
Aldolase	2.0 U/L	1.0-7.5 U/L
Lactate dehydrogenase (LDH)	1805 U/L	230-460
Albumin	2.7 g/dL	3.8-4.4
Uric acid	4.8 mg/dl	2.0-6.0
Calcium	9.1 mg/dl	8.1-10.4
Phosphorous	3.4 mg/dl	2.5-4.8
Magnesium	1.5 mg/dl	1.3-2.5
Rheumatoid factor	42 IU/ml	<15
Anti-citrullinated peptide (CCP)	7 U/ml	<17
Anti-tissue transglutaminase antibody (IgG, IgA)	Normal	-
Anti-nuclear antibody (ANA)	+2 speckled	-
Anti-smooth muscle antibody (ASMA)	Negative	-

The patient was started on prednisone at 1 mg/kg alongside azathioprine at 50 mg/day. Her myopathy showed some signs of improvement after two weeks of therapy and EMG changes reflected the improved muscle function. There was also a marked reduction noted in the CPK levels at one month. However, it was long before she gained functional use of her limbs again.

## Discussion

ASS is a rare autoimmune disorder with heterogenous clinical manifestations overlapping with those of other rheumatoid diseases. Different patients may present with distinct clinical patterns and the symptoms and signs may develop or change gradually over the course of the disease, thus, making it difficult to timely diagnose and manage patients with this condition [5]. Its most common clinical features include interstitial lung disease, myositis, and arthritis. Our patient presented with fever and night sweats, weight loss, myositis, arthralgia, and mechanic’s hands. She was misdiagnosed for two years leading to disease progression and significant morbidity due to severe myositis. A clear understanding of myalgias and myopathy among primary care physicians is therefore important to prevent confusion with non-specific symptoms such as fatigue. Mechanic’s hands are described as bilateral thick, cracked skin on the palms and radial surfaces of the digits due to hyperkeratosis and scaling [7]. Similar findings can also be appreciated on the soles of the patients in 90% of cases, called hikers’ feet [8]. These characteristic mechanic’s hands were ignored by the patient’s primary care physician and she was managed for fever with antipyretics, antihistamines, and multiple short courses of antibiotics over this period. It is imperative to obtain a detailed history and perform a detailed clinical examination to diagnose mechanic's hands and differentiate it from physiologic hyperkeratosis [8]. Our patient did not have any signs or symptoms of interstitial lung disease; however, it is important to obtain a chest X-ray and high-resolution computed tomography (HRCT) to avoid missing subclinical diseases and to differentiate the ASS-associated interstitial lung disease from other causes of idiopathic interstitial pneumonia [9].

Although the patient was investigated for autoimmune disorders previously, a substandard laboratory reported a negative ANA, highlighting the unequal access to standardized diagnostic services in our region. Similarly, in a case discussed by Rojas-Serrano et al., a 60-year-old lady faced a diagnostic delay of 20 years contributing to morbidity and significant progression of the disease [10]. Awareness regarding the disease manifestations, early referral to appropriate specialty, and availability of proper diagnostic modalities will contribute to prompt diagnosis and management of this disease [11]. A good interaction between community and hospital physicians, telemedicine, and monthly discussion of rare cases can be a good way to create awareness among primary care physicians regarding diagnostic dilemmas of rare and misdiagnosed diseases.

Diagnosis of the ASS can be aided by the definitive criteria provided by Connors et al. in 2010 and Solomon et al. in 2011, as shown in Table 2 [12,13].

**Table 2:** Diagnostic criteria for anti-synthetase syndrome according to Connors et al. and Solomon et al. ARS: aminoacyl-tRNA synthetase autoantibody.

Connors et al. diagnostic criteria [12]	Solomon et al. diagnostic criteria [13]
Definitive: the presence of ARS autoantibody	Definitive: the presence of ARS autoantibody
PLUS, one or more of the following:	PLUS, two major or one major and two minor criteria
Interstitial lung disease	Major:
Arthritis	Interstitial lung disease
Raynaud’s phenomena	Dermatomyositis or polymyositis by Bohan and Peter criteria
Fever	Minor:
Mechanic’s hands	Arthritis
	Fever
	Mechanic’s hands

The detection of ARS antibodies is crucial for both the Connors and Solomon criteria but their absence does not exclude the diagnosis of ASS because antibody titers may fluctuate with disease activity. The detection of the antibodies also depends upon the laboratories where they are being tested [7]. The presence and degree of myositis can be assessed by the elevated creatine kinase (CK) and serum aldolase levels [7]. The former was extremely raised in our case indicating that the disease had progressed rapidly during the time the patient had remained undiagnosed. Muscle biopsy is not routinely indicated in ASS [7].

No specific guidelines are available on the management of ASS due to the paucity of data and frequently misdiagnosed cases [12]. The treatment, however, includes anti-inflammatory and immunosuppressive drugs. Corticosteroids are the first-line agents with immunosuppressants such as azathioprine, mycophenolate mofetil, tacrolimus, rituximab, and cyclophosphamide as an add-on therapy for pulmonary and muscle involvement [7]. Our patient responded to steroids and azathioprine and did not need any additional initial therapy. The disease progression and remission are monitored with CK, aldolase activity, and periodic chest X-rays, and HRCT to monitor the muscle and lung disease [14].

The prognosis of ASS is based on prompt diagnosis followed by the commencement of appropriate immunosuppressive agents and periodic monitoring to prevent drug toxicity and ensure continuing efficacy of treatment [15].

## Conclusions

Our case calls attention to the lack of awareness of ASS among primary care physicians and unequal access to standard diagnostic services among our population, leading to late referral to specialists and contributing to the progression of the illness and the need for high doses of immunosuppressants to control the symptoms. The early recognition of the syndrome based on history, examination, laboratory, and radiological investigations is crucial to promptly initiate treatment to reduce the need for prolonged rehabilitation for myositis and lung transplantation and to increase survival. There is a need for more prospective studies to formulate guidelines on this topic to expedite diagnosis and management.
